# A potential anti-tumor effect of leukotriene C_4_ through the induction of 15-hydroxyprostaglandin dehydrogenase expression in colon cancer cells

**DOI:** 10.18632/oncotarget.16591

**Published:** 2017-03-27

**Authors:** Lubna M. Mehdawi, Shakti Ranjan Satapathy, Annika Gustafsson, Kent Lundholm, Maria Alvarado-Kristensson, Anita Sjölander

**Affiliations:** ^1^ Cell and Experimental Pathology, Department of Translational Medicine, Lund University, Skåne University Hospital, Malmö, Sweden; ^2^ Department of Surgery, Institute of Clinical Sciences, Sahlgrenska Academy, University of Gothenburg, Gothenburg, Sweden; ^3^ Molecular Pathology, Department of Translational Medicine, Lund University, Malmö, Sweden

**Keywords:** CysLTR2, 15-PGDH, colon cancer, LTC_4_ signaling, anti-tumor

## Abstract

Colorectal cancer (CRC) is one of the leading causes of cancer-related deaths worldwide. Cyclooxygenase-2, which plays a key role in the biosynthesis of prostaglandin E_2_ (PGE_2_), is often up-regulated in CRC and in other types of cancer. PGE_2_ induces angiogenesis and tumor cell survival, proliferation and migration. The tumor suppressor 15-hydroxyprostaglandin dehydrogenase (15-PGDH) is a key enzyme in PGE_2_ catabolism, converting it into its inactive metabolite 15-keto-PGE_2_, and is often down-regulated in cancer. Interestingly, CRC patients expressing high levels of the cysteinyl leukotriene 2 (CysLT_2_) receptor have a good prognosis; therefore, we investigated a potential link between CysLT_2_ signaling and the tumor suppressor 15-PGDH in colon cancer cells.

We observed a significant up-regulation of 15-PGDH after treatment with LTC_4_, a CysLT_2_ ligand, in colon cancer cells at both the mRNA and protein levels, which could be reduced by a CysLT_2_ antagonist or a JNK inhibitor. LTC_4_ induced 15-PGDH promoter activity via JNK/AP-1 phosphorylation. Furthermore, we also observed that LTC_4_, via the CysLT_2_/JNK signaling pathway, increased the expression of the differentiation markers sucrase-isomaltase and mucin-2 in colon cancer cells and that down-regulation of 15-PGDH totally abolished the observed increase in these markers.

In conclusion, the restoration of 15-PGDH expression through CysLT_2_ signaling promotes the differentiation of colon cancer cells, indicating an anti-tumor effect of CysLT_2_ signaling.

## INTRODUCTION

Colorectal cancer (CRC) is considered a major health problem worldwide [1]. It is the third most common cancer and the third most common cause of cancer-related deaths in both sexes [2]. Previous studies have shown that lifestyle habits, such as the excessive consumption of alcohol, red meat and processed food, a diet that is rich in fat or lacks fiber, and smoking are important risk factors for developing colorectal cancer [3]. Another important risk factor for CRC is chronic inflammation, particularly inflammatory bowel disease (IBD). CRC that develops due to IBD is the most common cancer among IBD patients [4].

Eicosanoids are biologically active lipophilic molecules derived from arachidonic acid (AA) that play an essential role in many physiological processes, such as inflammation [5]. These compounds are precursors to many different types of pro-inflammatory lipid mediators, such as leukotrienes (LTs) and prostaglandins (PGs), which are important regulators of the tumor microenvironment [5–6]. The cysteinyl leukotrienes (cysteinyl-LTs) LTC_4_, LTD_4_, and LTE_4_ are derived from AA via the 5-lipoxygenase (5-LOX) pathway. Cysteinyl-LTs play an important role in the pathogenesis of several chronic inflammatory diseases, such as IBD [7]. Cysteinyl-LT-mediated signaling is initiated by the binding of a ligand to one of two G protein-coupled receptors: CysLT_1_, which has a high affinity for LTD_4_, or CysLT_2_, which is the high-affinity receptor for LTC_4_ [8–9]. We have previously shown that the CysLT_1_ receptor is up-regulated in colorectal cancer patients and is associated with poor prognosis, while patients with high CysLT_2_ expression have a better prognosis [10]. Interestingly, a recent study revealed that both LTD_4_ and PGE_2_ increase the number of colon cancer-initiating cells, thereby initiating tumor growth in a xenograft mouse model [11]. Furthermore, in double-mutant *Cysltr1^-/-^/Apc^Min/+^* mice, a significant reduction of the tumor burden was observed compared to control littermates, and this effect was accompanied with decreased systemic inflammation indicated by PGE_2_ levels [12]. PGs, another important type of eicosanoid, are produced via the COX-2 pathway. COX-2 expression is typically absent in most cells and tissues under normal conditions; however, its expression is up-regulated during inflammation and in many cancers, including colon cancer [5]. Up-regulation of COX-2 in colorectal cancer increases the level of PGE_2_, which can induce most of the hallmarks of cancer by promoting proliferation, angiogenesis, survival, migration and invasion [13]. Recent epidemiological studies have indicated that the long-term use of non-steroidal anti-inflammatory drugs (NSAIDs) can decrease the incidence of certain malignancies, including colorectal, breast, lung and bladder cancers, by reducing prostanoid production through the inhibition of COX activity [5, 14].

The cytoplasmic enzyme 15-hydroxyprostaglandin dehydrogenase (15-PGDH) is the enzyme responsible for the degradation of PGE_2_, converting it into an inactive metabolite [15]. 15-PGDH is highly expressed in the normal colon mucosa, but it is lost in many CRCs [16], which is correlated with increased tumor formation [17–18]. Myung and coworkers showed that the deletion of the 15-PGDH gene *HPGD* increases colonic PGE_2_ levels and enhances tumorigenesis *in vivo*, demonstrating the tumor suppressor effect of 15-PGDH [16]. Gustafsson et al. showed that indomethacin, an NSAID, up-regulates 15-PGDH and at least partly decreases the expression of COX-2 in HCA-7 colon cancer cells [19]. Taken together, these data indicate that the levels of the pro-tumorigenic PGE_2_ are increased in CRC, which was previously attributed to the increased production via COX-2 up-regulation but more recently has also been attributed to decreased catabolism, reflecting the down-regulation of 15-PGDH.

We have previously shown that high CysLT_2_ receptor expression is correlated with a good prognosis in patients with colorectal cancer [10]. In the present study, we investigated the potential association between CysLT_2_ signaling and 15-PGDH expression in the pathogenesis of CRC.

## RESULTS

### The expression of COX-2, 15-PGDH and CysLT_2_ in colon cancer patients

**Figure 1:**
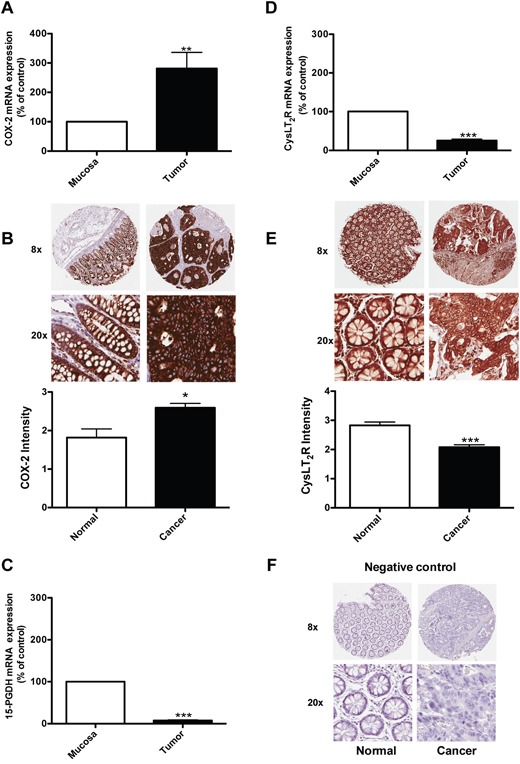
Expression of COX-2, 15-PGDH, and the CysLT2 receptor in colon cancer patients Quantification of the mRNA expression of **(A)** COX-2, **(C)** 15-PGDH, and **(D)** the CysLT_2_ receptor from 23 colon cancer patients by qPCR. **(B, E)** Immunohistochemistry staining with an antibody against the COX-2 (**(B)**, 1:200 dilution) or the CysLT_2_ receptor (**(E)**, 1:50 dilution); representative protein expression images from colorectal adenocarcinoma tissue and matched normal control tissue from one patient are shown. **(F)** Showing representative negative control immunohistochemistry staining. The intensity of the CysLT_2_ staining from all 22 patients was scored and presented in the graph. The data are presented as the percent of control (mucosa) and represent the mean ± standard error of mean (SEM). Statistical analysis was performed using an unpaired t-test; *P≤0.05, **P<0.01, ***P<0.001.

15-PGDH regulates the level of PGE_2_ produced through COX-2 and is a tumor suppressor frequently down-regulated in cancers [17, 20]. Here, we investigated the mRNA levels of COX-2, 15-PGDH, and the CysLT_2_ receptor in 23 colon cancer patients at different stages of the disease and compared the gene expression in normal mucosa and tumor tissues in matched pairs for each patient (Figure 1). Figure 1A shows higher levels of COX-2 expression in the tumor tissue compared to the normal mucosa area in colon samples from the same patients. Figure 1C and 1D shows that the expression levels of 15-PGDH and CysLT_2_ are significantly reduced in the tumor tissue of the patients compared to the normal mucosa area of the colon. Figure 1B and 1E shows the results from an immunohistochemistry analysis of carcinoma and normal tissues from 22 patients with CRC. The paired carcinoma and normal tissues were obtained from the same patient and stained with a specific CysLT_2_ receptor antibody or a COX-2 antibody. Higher levels of COX-2 expression in the tumor tissue compared with the corresponding normal tissue was observed (Figure 1B). We observed significantly lower CysLT_2_ staining in the tumor tissue compared with the corresponding normal tissue from the same patient. In a recent study, these paired samples were also stained with a 15-PGDH-specific antibody, and the normal colon samples showed significantly higher 15-PGDH protein expression compared to the matched colon cancer samples [21]. These findings are in good agreement with our mRNA data. We further confirmed the previous findings that COX-2 exhibits higher expression in tumor areas and that both the CysLT_2_ receptor and 15-PGDH are expressed at higher levels in normal areas compared to tumor areas in matched sample pairs from patients with colon cancer.

### The pro-inflammatory mediator LTC_4_ induces the up-regulation of 15-PGDH via the CysLT_2_ receptor in HT-29 colon cancer cells

**Figure 2:**
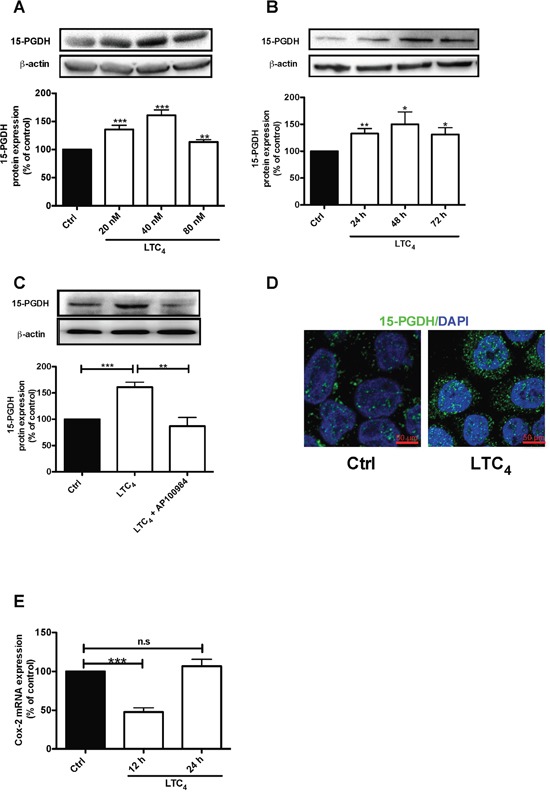
LTC4 up-regulates both the protein and mRNA levels of 15-PGDH in HT-29 cells **(A)** Western blot and densitometric analyses of LTC_4_-induced 15-PGDH protein expression. Cells were treated with 20, 40 or 80 nM LTC_4_ for 24 h, and the up-regulation of 15-PGDH was detected using a 15-PGDH antibody (1:5000 dilution). **(B)** Western blot and densitometric analyses of LTC_4_-induced 15-PGDH up-regulation after the cells were stimulated with 40 nM LTC_4_ for the indicated periods of time. **(C)** The cells were treated with 1 μM AP100984 (CysLT_2_ receptor antagonist) for 30 min prior to stimulation with or without 40 nM LTC_4_ for 24 h. The cells were lysed, subjected to SDS-PAGE and immunoblotting with a 15-PGDH antibody and subsequently re-incubated with an antibody against β-actin (1:1000 dilution) to ensure equal loading. **(D)** Confocal microscopy immunofluorescence images showing the expression of 15-PGDH, with antibody dilution of 1:200 (15-PGDH is shown in green; DAPI is in blue and was used at a 1:1000 dilution), after 24 h of stimulation with LTC_4_ in HT-29 cells. The objective used was 63x, and the scale bar is 50 μm. **(E)** mRNA analysis of the effect of LTC_4_ on COX-2 mRNA after 12 or 24 h of stimulation. The data are presented as the percent of untreated control cells and represent the mean ± SEM of at least three separate experiments. Statistical analysis was performed using an unpaired t-test; *P≤0.05, **P<0.01, ***P<0.001.

We previously showed that patients expressing high levels of CysLT_2_ have a good prognosis and higher long-term survival rates [22–23]. Myung and Yan confirmed the tumor suppressor activity of 15-PGDH through the restoration of 15-PGDH expression in human colon cancer cells, which blocks the ability of these cells to form tumors in nude mice hosts, and showed that knocking out the murine 15-PGDH gene markedly sensitizes mice to colon tumor development [16, 18]. Here, we investigated whether 15-PGDH is affected by LTC_4_ in HT-29 colon cancer cells. The results showed that LTC_4_ induced a significant increase in 15-PGDH protein expression (Figure 2A), with the most pronounced effect observed with 40 nM LTC_4_. LTC_4_ induced a significant up-regulation of 15-PGDH at the three time points examined, 24, 48 and 72 h (Figure 2B), which could be blocked by pretreatment with the CysLT_2_ receptor antagonist AP100984 (Figure 2C). Accordingly, these results were confirmed by confocal microscopy, which revealed LTC_4_-induced 15-PGDH up-regulation in these cells (Figure 2D). These results suggest that LTC_4_ could induce 15-PGDH expression via CysLT_2_. We next investigated the effect of LTC_4_ on *COX-2* mRNA and observed significant down-regulation after 12 h of stimulation with LTC_4_ (Figure 2E). This finding is interesting, as COX-2 is the enzyme responsible for the production of PGE_2_.

### LTC_4_ induces 15-PGDH promoter activity via JNK phosphorylation

**Figure 3:**
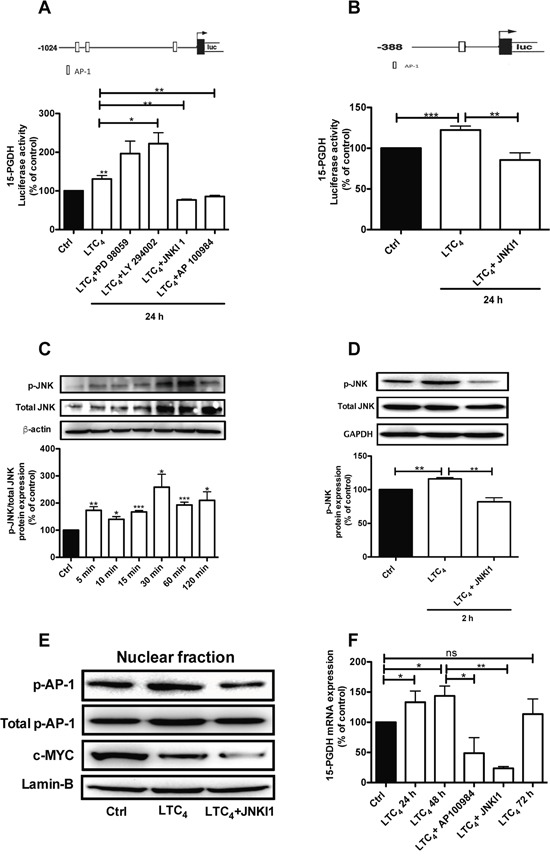
LTC4 induced 15-PGDH promoter activity in HT-29 cells **(A)** Schematic cartoon of both 15-PGDH promoters showing the different potential binding sites for the transcription factor AP-1 and the luciferase activity of 15-PGDH in cells seeded onto 12-well plates, transfected with the 15-PGDH promoter construct (-1024 bp) for 24 h, and subsequently pretreated with or without 50 μM PD 98059, 50 μM LY294002, or 10 μM JNK inhibitor I for 30 min prior to stimulation with 40 nM LTC_4_ for 24 h. **(B)** The cells were transfected with a 15-PGDH promoter construct (-388 bp) and pretreated with or without 10 μM JNK inhibitor I for 30 min prior to stimulation with 40 nM LTC_4_. **(C)** Western blot and densitometric analyses of LTC_4_-induced JNK phosphorylation in HT-29 cells at the indicated time-points. **(D)** The cells were treated with JNK inhibitor I 30 min prior to stimulation with or without 40 nM LTC_4_ for 2 h and then lysed, and the lysates were subjected to SDS-PAGE and immunoblotting with an antibody against p-JNK. The membrane was re-probed with an antibody against total JNK (both the p-JNK and total JNK antibodies were diluted 1:1000) and subsequently incubated with an antibody against GAPDH to ensure equal loading. **(E)** Western blot analyses of nuclear fractions of HT-29 cells incubated with JNK inhibitor I 30 min prior to stimulation or not with LTC_4_; the blots show phosphorylated AP-1 (AP-1; re-probed for total AP-1/Jun; both diluted 1:1000) and c-MYC (1:1000) and were re-probed for Lamin-B expression to ensure equal loading. **(F)** Analysis of the effect of LTC_4_ on 15-PGDH mRNA expression in HT-29 cells pretreated with or without the CysLT_2_ antagonist AP100984 or JNK inhibitor I. The data are presented as the percent of untreated control cells and represent the mean ± SEM of at least three separate experiments. Statistical analysis was conducted using an unpaired t-test and ANOVA; *P≤0.05, **P<0.01, ***P<0.001.

To verify the above findings, we next analyzed whether LTC_4_ could also induce 15-PGDH promoter activity. The results showed that LTC_4_ could induce 15-PGDH promoter activation and that this activation could be inhibited by AP100984, the CysLT_2_ antagonist (Figure 3A). To elucidate the potential signaling pathway by which LTC_4_ could regulate 15-PGDH expression, we used two different 15-PGDH promoter constructs that have different numbers of AP-1 binding sites (Figure 3A, 3B). Cells were transfected with the 15-PGDH promoter construct (-1024 bp) for 24 h and then pretreated or not with different pathway inhibitors, including PD 98059 (a selective MAP kinase inhibitor), LY 294002 (a PI3K inhibitor), and JNKI1, for 30 min prior to stimulation with or without 40 nM LTC_4_ for 24 h. We observed that the inhibition of the Erk1/2 or PI3K pathways did not block 15-PGDH promoter activity, while the inhibition of the JNK pathway blocked 15-PGDH promoter activation (Figure 3A), which indicates that the JNK pathway is a downstream target of the LTC_4_/CysLT_2_ signaling pathway. We further confirmed these results using a shorter 15-PGDH promoter sequence (-388 bp) that has only one AP-1 binding site (Figure 3B). For further investigation, we analyzed the c-Jun N-terminal kinase (JNK) by stimulating the cells with LTC_4_ for different time points and performing western blot experiments using a phospho-specific JNK antibody to observe the phosphorylation of JNK by LTC_4_. JNK phosphorylation was rapid and remained detectable after 2 h of stimulation, and this activation could be inhibited in the presence of JNK inhibitor I (Figure 3C and 3D). Next, we investigated whether the LTC_4_ signaling-induced activation of JNK is involved in the regulation of 15-PGDH based on the fact that the 15-PGDH promoter has binding sites for AP-1, which can be regulated through JNK. We observed that LTC_4_ elevated the phosphorylation of AP-1 in the nuclear fraction of the cells. The p-c-Jun/AP-1 antibody used detects phosphorylation at Ser73, which is needed for its transcriptional activity. Furthermore, this activation was significantly reduced in the presence of JNK inhibitor I, indicating the crucial role of JNK/AP-1 in the activation process that acts via LTC_4_ (Figure 3E). The reduced expression of c-MYC also confirmed the anti-tumor effect of LTC_4_ as previously described [22–23]. Furthermore, we also observed a significant induction of 15-PGDH at the mRNA level 24 and 48 h after LTC_4_ stimulation, which could also be inhibited through either AP100984 or JNK inhibitor I (Figure 3F).

### The pro-inflammatory mediator LTC_4_ induces the up-regulation of 15-PGDH via CysLT_2_ in Caco-2 colon cancer cells

**Figure 4:**
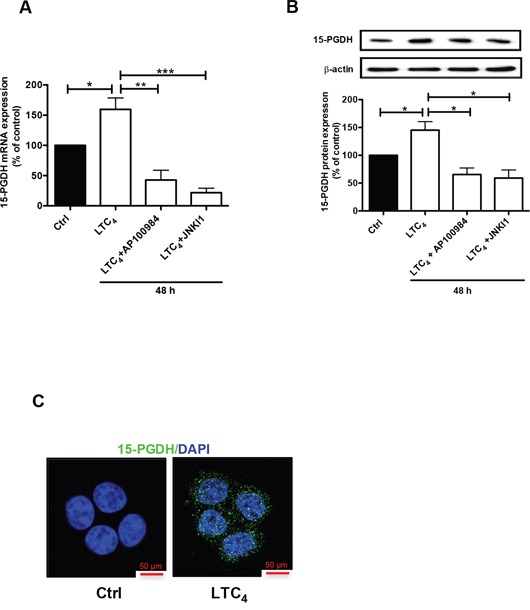
Effect of LTC4 on 15-PGDH expression in the colon cancer cell line Caco-2 **(A)** Quantification by qPCR of the mRNA expression of 15-PGDH following treatment with 40 nM LTC_4_ for 48 h in the presence or absence of AP100984 (a CysLT_2_ receptor antagonist, 1 μM) and JNK inhibitor I (10 μM). **(B)** Western blot showing 15-PGDH expression (antibody dilution, 1:5000) and densitometric analysis of LTC_4_-induced 15-PGDH up-regulation before or after the cells were stimulated with 40 nM LTC_4_ in the presence or absence of AP100984 and JNK inhibitor I for 48 h. **(C)** Confocal microscopy immunofluorescence images showing the expression of 15-PGDH (in green; antibody dilution, 1:200; DAPI in blue) after 24 h of stimulation with LTC_4_ in Caco-2 cells. The objective used was 63x. The data are presented as the percent of untreated control cells and represent the mean ± SEM of at least three separate experiments. Statistical analysis was conducted using an unpaired t-test; *P≤0.05, **P<0.01, ***P<0.001.

To confirm these results, we also investigated the effect of LTC_4_ on 15-PGDH expression in an additional colon cancer cell line, Caco-2. Similar to its effect in HT-29 cells, LTC_4_ could also induce the expression of 15-PGDH in Caco-2 cells, and this induction could be inhibited by AP100984 and JNK inhibitor I at both the mRNA (Figure 4A) and protein (Figure 4B) levels. We also confirmed that LTC_4_ induced the expression of 15-PGDH in Caco-2 cells using confocal microscopy (Figure 4C).

### CysLT_2_/JNK signaling induces CRC cell differentiation

Differentiation plays an important role in normal physiology. Cancer cells have a poor differentiation capacity, reflecting their proliferative nature. Independently, both CysLT_2_ and 15-PGDH inhibit the proliferation of colon and breast cancer cells [22–24]. Here, we investigated their roles in differentiation by studying two key terminal differentiation markers, Sucrase-Isomaltase (SI) and Mucin-2 [25].

**Figure 5:**
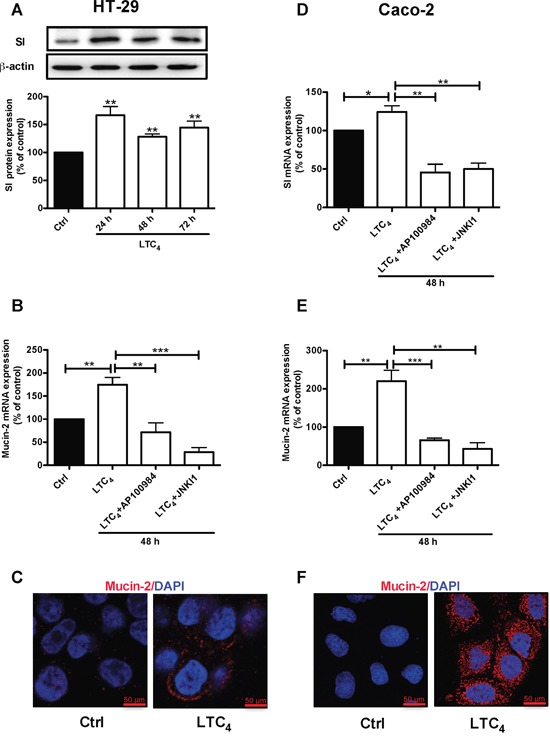
Effect of LTC_4_ on the expression of the differentiation markers SI and Mucin-2 in colon cancer cells **(A)** Western blot and densitometric analyses of SI expression after stimulation with 40 nM LTC_4_ for 24, 48 and 72 h in HT-29 cells. The SI antibody dilution was 1:1000. **(B)** Quantification by qPCR of the mRNA expression of Mucin-2. HT-29 cells were stimulated with 40 nM LTC_4_ after pretreatment with AP100984 (1 μM) or JNK inhibitor I (JNKI1, 10 μM) 30 min prior to stimulation. **(C)** Confocal microscopy immunofluorescence images showing the expression of Mucin-2 after 48 h of stimulation with LTC_4_ in HT-29 cells. **(D and E)** Quantification by qPCR of SI and Mucin-2 mRNA expression in Caco-2 cells. Caco-2 cells were stimulated with 40 nM LTC_4_ and pretreated with AP100984 and JNKI1 30 min prior to stimulation for 48 h. **(F)** Confocal microscopy immunofluorescence images showing the expression of Mucin-2 (C, F in red; antibody dilution, 1:500; DAPI in blue dilution 1:1000) after 48 h of stimulation with LTC_4_ in Caco-2 cells. The objective used was 63x, and the scale bar is 50 μm. The data are presented as the percent of untreated control cells and represent the mean ± SEM of at least three separate experiments. Statistical analysis was conducted using an unpaired t-test and ANOVA; *P≤0.05, **P<0.01, ***P<0.001.

**Figure 6:**
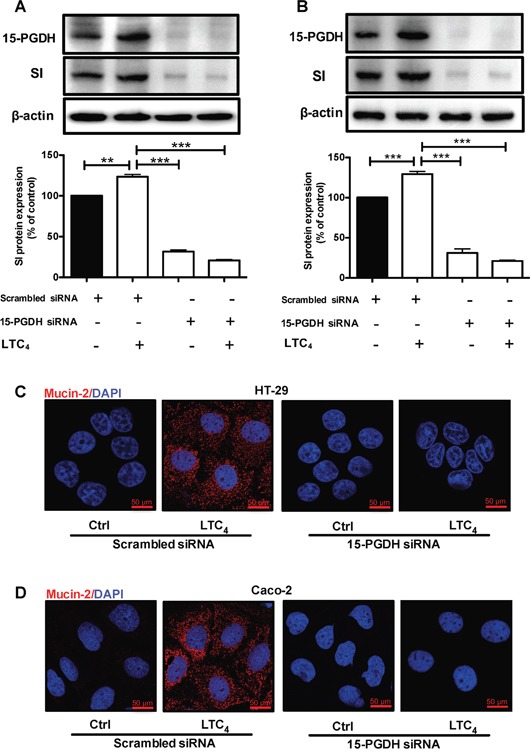
Effect of 15-PGDH down-regulation on the cell differentiation markers SI and Mucin-2 **(A)** A representative western blot and densitometric analysis of LTC_4_-induced SI protein expression after transfection with a scrambled control siRNA or 15-PGDH siRNA in HT-29 cells are shown. The cells were treated with or without 40 nM LTC_4_ for 48 h, and the change in the SI protein level was detected using an SI-specific antibody (1:1000 dilution) and 15-PGDH was detected using a 15-PGDH antibody (1:5000 dilution). The membrane was re-probed with an antibody against β–actin to ensure equal loading. **(B)** A representative western blot and densitometric analysis performed as in **(A)** shown here for Caco-2 cells. **(C, D)** Representative confocal microscopy immunofluorescence images from cells transfected with a scrambled control siRNA or 15-PGDH siRNA with or without stimulation with 40 nM LTC_4_ for 48 h. The expression of Mucin-2 (in red; antibody dilution, 1:500; DAPI in blue, dilution 1:1000) **(C)** in HT-29 cells and **(D)** Caco-2 cells is shown. The objective used was 63x, and the scale bar is 50 μm. The data are presented as the percent of control cells and represent the mean ± SEM of at least three separate experiments. Statistical analysis was conducted using an unpaired t-test; *P≤0.05, **P<0.01.

HT-29 cells were treated with 40 nM LTC_4_ for 72 h, and western blotting was performed using a specific SI antibody. A significant up-regulation of the SI protein level was observed at all time points (24, 48 and 72 h; Figure 5A). To investigate whether the LTC_4_ signaling axis mediated the increase in Mucin-2 at the transcriptional level, we examined Mucin-2 mRNA levels in HT-29 cells after 48 h of treatment with LTC_4_ in the presence of either AP100984 or JNK inhibitor I. LTC_4_ significantly increased Mucin-2 mRNA expression, which showed a nearly two-fold up-regulation at 48 h, and this increase was significantly decreased in the presence of AP100984 or JNK inhibitor I (Figure 5B). In parallel experiments, we further showed the effect of LTC_4_ on the expression of Mucin-2 through immunofluorescence analysis (Figure 5C). Moreover, Caco-2 cells were treated with or without 40 nM LTC_4_ in the presence or absence of AP100984 or JNK inhibitor I for 48 h to investigate the expression of the differentiation markers SI and Mucin-2 at the mRNA level. In HT-29 cells, LTC_4_ significantly induced the expression of SI and Mucin-2. This effect could be inhibited in the presence of the CysLT_2_ antagonist AP100984 and JNK inhibitor I also in Caco-2 cells (Figure 5D and 5E). Confocal microscopy revealed that similar to HT-29 cells, LTC_4_ could induce the expression of the Mucin-2 protein in Caco-2 cells (Figure 5F). We next knocked down the expression of 15-PGDH using siRNA. Our results clearly show that the 15-PGDH siRNA reduced the 15-PGDH and SI protein levels and totally abolished the LTC_4_-induced increase in 15-PGDH and SI after 48 h of stimulation compared to a scrambled control siRNA in both HT-29 cells (Figure 6A) and Caco-2 cells (Figure 6B). In a similar set of experiments, we analyzed the Mucin-2 protein expression by confocal microscopy. We found that down-regulation of 15-PGDH totally abolished the ability of LTC_4_ to induce Mucin-2 expression in both HT-29 cells (C) and Caco-2 cells (D). Taken together, these results suggest that LTC_4_ signaling induces the differentiation of colon cancer cells via the CysLT_2_/JNK/15-PGDH pathway.

## DISCUSSION

15-PGDH is a tumor suppressor, and the loss of this protein has been observed in many cancers, such as breast, colon and gastric carcinoma [20, 26–27]. The re-expression of tumor suppressor proteins in cancer has been shown to be a promising therapeutic strategy [15]. We have previously shown that colorectal cancer patients with high levels of CysLT_2_ have good prognoses [10]. In addition, we have also shown that LTC_4_ could reduce the migration of breast cancer cells and that CysLT_2_ signaling could contribute to the ATRA-induced differentiation of colon cancer cells [22–23]. Kim et al. showed marked blunted mucosal mast cell hyperplasia in the lungs of mice lacking leukotriene C_4_ synthase (LTC_4_ S), the terminal enzyme needed for cysteinyl-LT synthesis [28]. These findings strongly suggest that the activation of CysLT_2_ might have an anti-tumor effect.

In many cancers, including colon cancer, COX-2 is up-regulated, leading to PGE_2_ production, which is an important regulator of many effects including the processes involved in the hallmarks of cancer [13]. High levels of PGE_2_, a known contributor to the tumor microenvironment of CRC patients, have been previously attributed to increased synthesis through COX-2 up-regulation and more recently to decreased catabolism by 15-PGDH, which is down-regulated in many CRCs [29]. The findings of the present study showed that both mRNA and protein levels of COX-2 are increased in tumor tissue compared to normal mucosa from the same colon cancer patients, whereas the mRNA levels of 15-PGDH and CysLT_2_ are down-regulated. Using IHC, we previously showed that normal colon samples express high protein levels of 15-PGDH and that matched CRC patient tissues express low protein levels of 15-PGDH, and a similar pattern was also observed for CysLT*2* protein expression [10, 21].

Based on previous findings, we hypothesized that LTC_4_ could induce the expression of 15-PGDH through CysLT_2_. Our data showed that LTC_4_ significantly induced the expression of 15-PGDH at both the mRNA and protein levels in HT-29 and Caco-2 colon cancer cells. However, this effect could be inhibited by a CysLT_2_ antagonist (AP100984). Mann et al. reported that EGF, through the EGFR, a known inducer of cell proliferation and tumor cell invasion, can repress the activity of the 15-PGDH promoter by inducing SNAIL, which binds to a conserved E-box element in the PGDH promoter and represses transcription [30]. Another study showed that like CysLT_2_, butyrate, produced by commensal bacteria, can induce the differentiation of colon cancer cells through the activation of the transcription factor AP-1 [31]. Greenland et al. showed that the human NAD^+^-dependent 15-PGDH gene promoter is controlled through the transcription factors ETS and AP-1 in a number of different cell types of uterine, placental, and hematopoietic cancers [32]. We therefore investigated the potential effect of LTC_4_ on the regulation of 15-PGDH promoter activity. The results showed that LTC_4_ induced the phosphorylation of JNK via CysLT_2_ signaling. Activated JNK thereby activates AP-1, which subsequently binds to and activates the 15-PGDH promoter. Interestingly, the results of a recent study showed that WNT5A activates 15-PGDH via a JNK/AP-1-dependent pathway and induces the differentiation of colon cancer cells [21]. This finding is interesting, as β-catenin–dependent signaling down-regulates 15-PGDH expression, and WNT5A can counteract this signaling by reducing active β–catenin signaling and inducing JNK/AP-1 signaling. These data showed that LTC_4_ also induced the differentiation of both HT-29 and Caco-2 colon cancer cells, demonstrated through the up-regulation of two key differentiation markers, Sucrase-Isomaltase (SI) and Mucin-2, which was also observed after treatment with WNT5A in HT-29 cells [21]. We presumed that the up-regulation of these markers was mediated via a CysLT_2_/JNK/AP-1 signaling pathway since the induced expression of these proteins could be blocked by the CysLT_2_ antagonist AP100984 and JNK inhibitor I in both colon cancer cell lines. We therefore concluded that CysLT_2_ signaling could induce colon cancer cell differentiation. Furthermore, the fact that LTC_4_ signaling also reduced the expression of c-MYC is an interesting observation consistent with the proposed anti-tumor effect of LTC_4_ in colon cancer cells. Interestingly, we previously observed that high levels of the CysLT_1_ receptor are consistent with a poor prognosis, and in a xenograft model with colon cancer cells, CysLT_1_ receptor inhibition with the CysLT_1_ antagonist montelukast reduced the tumor burden in these mice, and treatment with montelukast resulted in reduced stemness in combination with reduced colony formation in colon cancer cells [10, 33–34]. Moreover, mice lacking a functional CysLT_1_ receptor in an APC^Min/+^ model have significantly reduced tumors/polyps in the intestine [12]. Taken together, these results suggest that the inhibition of CysLT_1_ can be beneficial for colon cancer patients by shifting signaling from the more oncogenic CysLT_1_ receptor to the more tumor-suppressing CysLT_2_ receptor.

**Figure 7:**
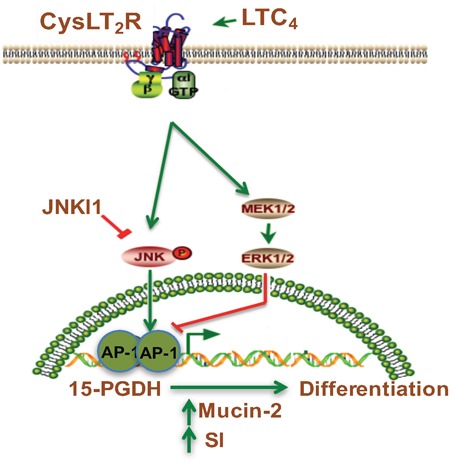
Schematic representation of the induction of 15-PGDH promoter activity by LTC4 via the CysLT2 receptor signaling pathway LTC_4_, leukotriene C_4_; CysLTR2, cysteinyl leukotriene 2 receptor; JNKI1, c-Jun N-terminal kinase (JNK) inhibitor; AP-1 transcription factor.

In conclusion, we provide the first evidence that LTC_4_, via CysLT_2_ signaling, can induce 15-PGDH expression through the activation of the JNK/AP-1 pathway (see Figure 7). This activation in turn induces the differentiation of colon cancer cells, while 15-PGDH down-regulation totally abolished the LTC_4_-induced increase in the expression of the differentiation markers Mucin-2 and SI. These data support the previous finding that the CysLT_2_ receptor might have an anti-tumorigenic effect in colon cancer.

## MATERIALS AND METHODS

### Cell lines

HT-29 colon adenocarcinoma cells (ATCC^®^ HTB-38™) were grown in McCoy's 5A modified medium (HyClone^™^, GE Healthcare Life Sciences), and Caco-2 colon adenocarcinoma cells (ATCC^®^ HTB-37™) were grown in DMEM (Sigma Life Science, St. Louis, MO, USA). All media were supplemented with 10% fetal bovine serum, 100 μg/ml penicillin/streptomycin and glutamine, and the cells were maintained at 37°C in a humidified atmosphere containing 5% CO_2_. All the supplements were procured from Sigma Life Science. The cells were regularly tested to ensure that they were free of mycoplasma infections and were subjected to STR profiling through the LGC Standards cell line authentication service.

### Patient samples

Tumor tissues and corresponding normal mucosa from 23 colon cancer patients were obtained from the Department of Clinical Science, Sahlgrenska Academy, Gothenburg, Sweden. Tumor and large bowel tissue samples were collected at the serosa level, fresh frozen in liquid nitrogen and stored at -80°C until further analysis. A certified pathologist staged all tumors. The tumor samples contained approximately 70-80% tumor cells based on visual inspection [35]. Matched tumor and normal tissue sample pairs obtained from 22 patients who underwent surgery for colorectal cancer at Malmö University Hospital during a selected time period in 1990 were mounted in TMA. These TMA samples have previously been described and included in a retrospective study [21]. The Ethical Committee at Lund University approved this study.

### Antibodies and reagents

The following antibodies and reagents were used: The rabbit polyclonal anti-15-PGDH antibody (NB200-179) was purchased from Novus Biologicals (Cambridge, UK), and the rabbit polyclonal anti-COX-2 (ab52237) antibody was obtained from Abcam (Cambridge, UK). The rabbit polyclonal antibody against sucrase-isomaltase (SAB2105150) and LY294002, a specific PI3K inhibitor, were purchased from Sigma Life Science (St. Louis, MO, USA). Cysteinyl leukotriene C_4_ was purchased from Larodan Fine Chemicals (Ann Arbor, MI, USA). The goat polyclonal Mucin-2 antibody (sc-23171) and the antibodies against p-JNK (sc-6254), total JNK (sc-571), lamin B (sc-6216) and GAPDH (sc-25778) were purchased from Santa Cruz Biotechnology (Santa Cruz, CA, USA). The CysLT_2_ receptor antagonist AP-100984 was a kind gift from Dr. J Evans, Amira Pharmaceuticals (San Diego, CA, USA). PD98059, a selective inhibitor of MAP kinase, was purchased from Cell Signaling Technology (Beverly, MA, USA). The cell permeable JNK inhibitor I (L-form, Cat. No. 420116) was purchased from Calbiochem (San Diego, CA, USA). The rabbit monoclonal antibodies against phosphor-c-Jun/AP-1 (Ser73, Cat. No. 3270) or total c-Jun/AP-1 (60A8) were procured from Cell Signaling Technology (Danvers, MA, USA). The secondary peroxidase-linked goat anti-rabbit and goat anti-mouse antibodies were from Dako (Glostrup, Denmark), and the enhanced chemiluminescent HRP substrate was obtained from Millipore (Merck KGaA, Darmstadt, Germany). The Alexa Fluor 488- and 568-conjugated secondary antibodies were from Molecular Probes/ThermoFisher Scientific (Waltham, MA, USA). Immun-blot^®^PVDF membranes were purchased from Bio-Rad (Hercules, CA, USA). The NE-PER™ nuclear and cytoplasmic extract reagent kit was obtained from Thermo Fisher Scientific (Waltham, MA, USA).

### Gel electrophoresis and immunoblotting

The cells were cultured for 5 days and stimulated with different concentrations of LTC_4_ for different lengths of time as indicated in the figures. After stimulation, the cells were washed two times with ice-cold 1X PBS and lysed with lysis buffer (50 mM Tris pH 7.5, 1 mM EDTA, 1 mM EGTA, 2 mM Na_3_VO_4_, 1% Triton X-100, 50 mM NaF, 5 mM sodium pyrophosphate, 10 mM β-glycerophosphate, 4 μg/ml Leupeptin, and 60 μg/ml PMSF) and incubated on ice for 1 h. Thereafter, the cells were homogenized 10 times using a syringe and centrifuged at 10,000 ×*g* for 10 min, and the supernatant was then collected. Nuclear extracts of treated and untreated cells were prepared using NE-PER™ according to the manufacturer's instructions. Whole cell lysates and nuclear lysate samples were adjusted for equal protein content by the Bradford method using Coomassie blue (Pierce, Rockford, IL, USA). The samples were boiled for 10 min in Laemmli sample buffer (0.5 M Tris-base, 10% SDS, glycerol, bromophenol blue, and 15 mg/ml DTT) and subsequently subjected to 10% SDS polyacrylamide gel electrophoresis (SDS-PAGE). Thereafter, the proteins were transferred to a PVDF membrane. The membrane was blocked for 1 h with 3% BSA in 1X PBS at RT and incubated with a primary antibody overnight at 4°C. The membrane was extensively washed with PBS-Tween and incubated with secondary antibody (goat anti-rabbit or goat anti-mouse, dilution 1:1000) for 1 h at RT. The membranes were incubated with a chemiluminescent HRP substrate for the detection of the immune-protein complexes using the Bio-Rad ChemiDoc XRS^+^ system. Bio-Rad Image Lab software was used for densitometric analysis, and the value obtained from the untreated (control) was set to 100.

### Real-time PCR (qPCR)

Untransfected and siRNA (control and 15-PGDH)-transfected HT-29 and Caco-2 cells were pre-incubated with or without AP100984 (1 μM) or JNKI I (10 μM) for 30 min before stimulation with 40 nM LTC_4_ for the indicated lengths of time, washed in PBS and immediately frozen at -80°C. Total RNA was isolated from the different cell and tissue samples using the Qiagen RNeasy Plus Mini Kit. The cDNA synthesis was performed using RevertAid H Minus M-MuLV reverse transcriptase (Thermo Scientific, USA). The following primers (from Applied Biosystems, Cambridge, UK) were used: *PTGS2* (Hs00153133_m1), *CYSLTR2* (Hs00252658_s1), *HPGD* (Hs00168359_m1), *SI* (Hs00356112_m1), *MUC2* (Hs00159374_m1), and *HPRT1* (Hs99999909_m1). The amplification reactions were performed on an Mx3005P system (Agilent Technologies, Inc., CA, USA). The data were normalized to the housekeeping gene HPRT1 and analyzed using MxPro qPCR software.

### siRNA transfection

Transient siRNA transfection of HT-29 and Caco-2 cells was performed according to the manufacturer's instructions. Briefly, 50% confluent cells were transfected with 100 nM siRNA (control or 15-PGDH) using Lipofectamine^®^2000 (ThermoFisher Scientific, USA) for 6-8 h in Opti-MEM with reduced serum and without antibiotics. The PGDH siRNA (sc-61330) and the controls siRNA-A, siRNA-B, and siRNA-C (sc-37007, sc-44230, sc-44231) were from Santa Cruz Biotechnology. The cells were allowed to rest for 48 h in complete culture medium. Thereafter, the cells were stimulated or not on day 5 after seeding.

### Transfection and luciferase assay

A Dual-Luciferase Reporter Assay System (Promega, Madison, WI, USA) was used for the following experiments. 15-PGDH promoter plasmids (a gift from Professor Birgit Gellersen, University of Hamburg, Germany) [31] were used at a final concentration of 1 μg/ml. The transfections were conducted in serum-free medium according to the manufacturer's instructions. Polyfect (Qiagen) was used as the transfection reagent (at a ratio of 4:1). The DNA concentration of the Renilla luciferase transfection control was 0.2 μg/ml. The cells were incubated for 24 h at 37°C after the transfections, and the medium was then replaced with serum-containing medium. After an additional 24 h, the cells were incubated for 2 h in serum-free medium prior to further incubation with or without AP100984, LY 294002, PD 98059 and JNKI1 for 30 min, followed by LTC_4_ (40 nM) stimulation for 24 h. Untreated cells were used as the control. The cells were lysed with passive lysis buffer and stored at -20°C. The thawed lysates were centrifuged for 5 min at 1000 *×g* and analyzed using the Dual-Luciferase Reporter Assay System from Promega according to the manufacturer's instructions. The luminescence of the firefly and Renilla luciferase was measured on a MiniLumat LB 9506 luminometer (Berthold Technologies GmbH, Dusseldorf, Germany) according to the protocol, and the ratio between the two was calculated. All samples were analyzed in triplicate for each experiment.

### Immunohistochemistry

TMA paraffin blocks were cut into 4 μm sections and pretreated as previously described [32]. All immunohistochemical procedures were performed using a Dako automatic slide stainer (Dako, Glostrup, Denmark) according to the manufacturer's instructions, and the sections were counterstained with H&E. Antibodies against the CysLT_2_ receptor (1:50) and COX-2 (1:200) were used. As a secondary antibody, we used an EnVision Flex Mini kit (prediluted, Dako autostainer); this dual link system detects primary mouse and rabbit antibodies and is also used as a negative control (see Figure 1F). The slides were scored for the intensity of each stained sample as 0 = negative, 1 = weak, 2 = intermediate and 3 = strong. All the stained slides were scored independently and in a blinded manner. All slides were scanned with the Aperio Scanscope XT System.

### Immunofluorescence analysis

HT-29 and Caco-2 cells were cultured on coverslips for 3 days and subsequently stimulated or not with LTC_4_ for 24 or 48 h. The same procedure was followed for siRNA-transfected cells. The cells were washed with PBS, fixed and permeabilized in methanol/acetone (1:1; -20°C for 4 min). The cells were washed with PBS and incubated for 30 min in PBS staining buffer (PBS, 1% FCS, and 0.5% BSA) to prevent non-specific antibody binding. The cells were incubated for 1 h with a primary antibody against 15-PGDH (1:200 dilution) or Mucin-2 (1:500 dilution), washed with PBS staining buffer, incubated for 1 h with an Alexa Fluor 488- or 568-conjugated secondary antibody (1:1000 dilution) and finally stained with DAPI for 3 min (1:1000). The coverslips were washed and mounted onto glass slides using Zeiss fluorescent mounting medium. Fluorescence images were captured using a Zeiss LSM 700 (Carl Zeiss Microscopy GmbH, Jena, Germany) confocal microscope.

### Statistical analysis

Prism software 5.0 (GraphPad software, San Diego, CA) was used for statistical analyses. Student's t-test and one-way ANOVAs were used, and statistical significance was determined as P ≤0.05. All means were calculated from the data obtained from at least three different experiments.

## Abbreviations

15-PGDH: 15-hydroxyprostaglandin dehydrogenase
CRC: colorectal cancer
COX-2: cyclooxygenase-2
Cysteinyl-LTs: cysteinyl leukotrienes
CysLT_2_: cysteinyl leukotriene receptor 2
LTs: leukotrienes
PGE_2_: prostaglandin E_2_
PGs: prostaglandins
